# Investigating morphological changes in the brain in relation to etiology and duration of olfactory dysfunction with voxel-based morphometry

**DOI:** 10.1038/s41598-021-92224-w

**Published:** 2021-06-16

**Authors:** E. M. Postma, P. A. M. Smeets, W. M. Boek, S. Boesveldt

**Affiliations:** 1 Division of Human Nutrition and Health, Wageningen University & Research, P.O. Box 17, 6700 AA Wageningen, The Netherlands; 2 ENT Department, Hospital Gelderse Vallei, Ede, Willy Brandtlaan 10, 6716 RP Ede, The Netherlands; 3 Image Sciences Institute, University Medical Center Utrecht Brain Center, Utrecht University, Heidelberglaan 100, 3584 CX Utrecht, The Netherlands

**Keywords:** Brain imaging, Magnetic resonance imaging

## Abstract

Olfactory loss (OL) affects up to 20% of the general population and is related to changes in olfaction-related brain regions. This study investigated the effect of etiology and duration of OL on gray matter volume (GMV) of these regions in 257 patients. Voxel-based morphometry was applied to measure GMV in brain regions of interest to test the effects of etiology and duration on regional GMV and the relation between olfactory function and regional GMV. Etiology of OL had a significant effect on GMV in clusters representing the gyrus rectus and orbitofrontal cortex (OFC), bilaterally. Patients with congenital anosmia had reduced GMV in the gyrus rectus and an increased OFC volume compared to patients with acquired OL. There was a significant association between volume of the left OFC and olfactory function. This implies that changes in GMV in patients with acquired OL are mainly reflected in the OFC and depend on olfactory function. Morphology of olfactory areas in the brain therefore seems to relate to olfactory function and the subsequent degree of exposure to olfactory input in patients with acquired OL. Differences in GMV in congenital anosmia are most likely due to the fact that patients were never able to smell.

## Introduction

Olfaction is important in daily life: odors do not only affect how food tastes, but play a role in many processes, such as the detection of danger, like smelling a fire or leaking gas, or in social situations. Although olfactory loss is not often discussed, 3% up to 20% of the general population is affected by olfactory loss^1–3^. Patients with olfactory loss report decreased quality of life, issues with daily safety and diminished appetite^4,5^. Olfactory loss can therefore have a major impact on daily life. The most common causes of olfactory loss are post-infectious loss, chronic rhinosinusitis and head trauma^6,7^. Currently, a new population of patients with olfactory loss is arising, as this is one of the symptoms of a Covid-19 infection^8^.

After entering the nose, odors stimulate the olfactory epithelium located at the roof of the nasal cavity. Through the olfactory nerve, the olfactory receptor cells in the epithelium first project to the olfactory bulb, which is the key odor processing structure, located at the base of the prefrontal cortex. A larger volume of the olfactory bulb is correlated with better olfactory function, in both healthy people^9,10^ and patients with olfactory loss^11,12^. Beyond the olfactory bulb, the piriform cortex, the entorhinal cortex and the amygdala are considered as primary olfaction-related areas^13^. Other regions that are known to play a role in odor processing, so-called secondary olfaction-related regions, are the orbitofrontal cortex, the anterior cingulate cortex and the insular cortex^13,14^.

The central olfactory system is plastic and is affected by peripheral olfactory input. Training of the olfactory system can lead to changes in morphology of olfaction-related regions, as has been shown in for example wine tasters or perfumers^15^. These experts train their sense of smell in a professional way, which can lead to increased volume of the right insula and entorhinal cortex^16^.

While these experts have an *improved* sense of smell due to their training, patients with olfactory loss are subject to decreased olfactory input. This might, similarly, induce morphological changes in olfaction-related regions of the brain. Previous studies demonstrated a decreased volume of the piriform cortex in patients with olfactory loss^17–20^. Additionally, evidence for a decrease in volume of the anterior cingulate cortex was found^17–21^, as well as the orbitofrontal cortex^17–19,21–23^ and the insular cortex^18–22^. Moreover, reduced olfactory input in patients with olfactory loss can lead to reorganization of neural processes^24,25^. Although olfactory loss is related to changes in olfaction-related brain regions, so far there are little studies that compared the morphology of olfaction-related brain regions in patients with olfactory loss by different etiologies or durations. However, it is known that these can affect outcomes, as duration of olfactory loss can predict recovery^26^ and volume of the olfactory bulbs is different between etiologies of olfactory loss^11^.

Most people with olfactory dysfunction lose their sense of smell during life, mostly due to disease or a head trauma, while some people are born without the sense of smell, so-called congenital anosmia^27^. Previous research showed an increase of gray matter volume in the piriform cortex in patients with congenital anosmia compared to healthy controls^28,29^. Findings on morphology of the orbitofrontal cortex are conflicting. Frasnelli et al. reported an increase of cortical thickness of the medial orbitofrontal cortex bilaterally, and Peter et al. found increased gray matter volume in the medial orbital gyrus bilaterally^29,30^. In contrast, Karstensen et al. found reduced gray matter volume in the left medial orbitofrontal cortex^28^. Moreover, hitherto research on the differences in brain morphology between patients with congenital anosmia and acquired olfactory loss is lacking.

Understanding the consequences of reduced exposure to olfactory input due to olfactory loss on the olfactory system in the brain can deepen our insight in the processes that underlie morphological changes (over time). Therefore, this study aimed to obtain more insight in the relation between olfactory loss and gray matter volume of olfaction-related brain areas. We included a clinical population of patients with olfactory loss, containing different etiologies and durations of olfactory loss. Moreover, we investigated the relation between olfactory function and gray matter volume of olfaction-related brain regions.

## Results

### Patient characteristics

In the patient population, 148 patients (57.6%) were functional anosmic (average TDI-score: 11.1 ± 2.72 ) and 99 patients (38.5%) were hyposmic (average TDI-score: 22.0 ± 4.38).

Patients with congenital anosmia were significantly younger than patients with any other etiology (χ^2^(3) = 49.49, *p* < 0.001). Olfactory function was significantly better in patients with post-infectious olfactory loss compared to patients with olfactory loss after chronic inflammation, idiopathic olfactory loss or congenital anosmia. Moreover, olfactory function was also significantly better in patients with olfactory loss after chronic inflammation and idiopathic olfactory loss compared to patients with congenital anosmia (χ^2^(3) = 43.76, *p* < 0.001). There was no significant effect of etiology on total gray matter volume (F(3,250) = 1.124, *p* = 0.340; Table 1).

**Table 1:** Demographics of patients who were included in the analysis, categorized on etiology and duration of olfactory loss.

	Age (years)	Males/females (n)	Years of olfactory loss					Sniffin’ Sticks score	Total gray matter volume (mm^3^)
			0–2	2–5	5–10	> 10	Congenital		
**Etiology (n = 257)**									
Post-infectious (n = 87)	61 ± 11.6^a^	26 / 61	41	27	8	11	0	18.6 ± 6.23^a^	570 ± 64
Chronic inflammation (n = 63)	59 ± 11.9^a^	39 / 24	9	8	20	26	0	14.8 ± 6.46^b^	584 ± 58
Idiopathic (n = 80)	62 ± 13.5^a^	38 / 42	10	31	19	20	0	14.3 ± 5.48^b^	573 ± 69
Congenital (n = 27)	33 ± 16.4^b^	12 / 15	0	0	0	0	27	10.1 ± 2.94^c^	634 ± 63
**Duration (n = 230)**									
0–2 years (n = 60)	61 ± 10.6	15 / 45	N/A					19.8 ± 6.43^a^	570 ± 60
2–5 years (n = 66)	62 ± 12.4	31 / 35	N/A					15.6 ± 5.16^b^	566 ± 68
5–10 years (n = 47)	59 ± 14.1	26 / 21	N/A					14.8 ± 6.26^b^	589 ± 67
> 10 years (n = 57)	61 ± 12.9	31 / 26	N/A					13.8 ± 5.98^b^	578 ± 62

Age, Sniffin’ Sticks scores and gray matter volumes are reported as mean ± SD. Rows for etiology or duration that do not share similar superscript letters (a or b) are significantly different. In case no letters are shown, there were no significant differences for that row.

Olfactory function was significantly better in patients with olfactory loss for 0–2 years compared to longer durations. There was no significant effect of duration on total gray matter volume (F(4,249) = 1.993, *p* = 0.096; Table 1).

### Effect of etiology on gray matter volume

**Figure 1:**
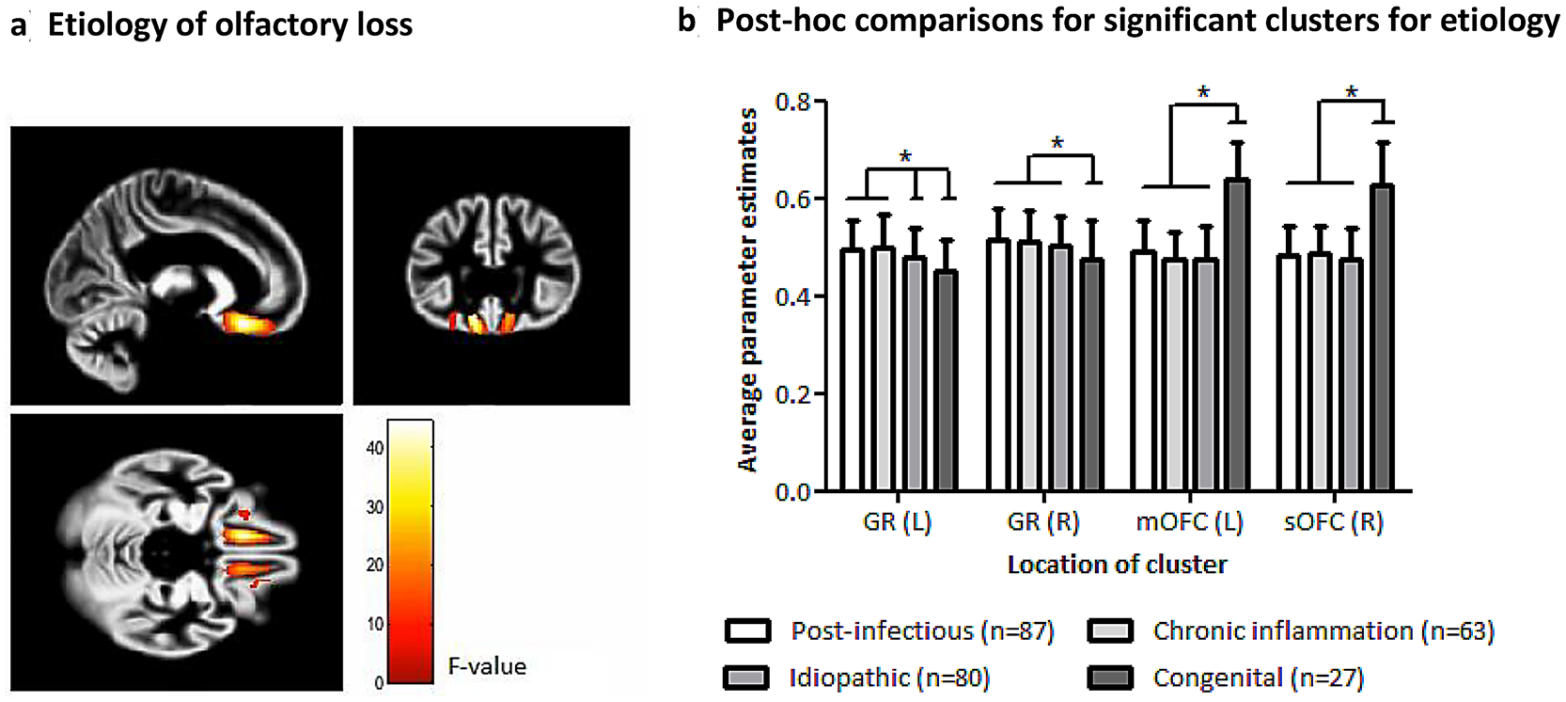
(**a**) Main effect of etiology group on ROI gray matter volume in the gyrus rectus (GR) and medial and superior orbitofrontal cortex (mOFC; sOFC), reported at *p* < 0.05 (FWE); results are shown as F-map overlaid on the gray matter group template; (**b**) Average gray matter volume per cluster per group, reported as mean ± SD; an asterisk indicates significant differences between groups.

The model for etiology of olfactory loss showed a main effect of etiology group on gray matter volume within the a priori defined ROIs, which was reflected in four significant clusters: two in the gyrus rectus and two in the orbitofrontal cortex (OFC), bilaterally (Table 2 and Fig. 1a). When comparing the different groups, post-hoc testing displayed that patients with congenital anosmia had reduced volume in the gyrus rectus compared to the other patient groups. In contrast, OFC volume in patients with congenital anosmia was increased compared to the other patient groups (Fig. 1b).

**Table 2:** Significant clusters from the F-test for a main effect of etiology of olfactory loss.

	Hemisphere	MNI coordinates (x y z)	Z-value (peak)	Cluster size (voxels)
Gyrus rectus	L	− 11 26 − 20	> 8.00	941
Gyrus rectus	R	12 24 − 17	7.43	694
Superior orbitofrontal cortex	R	20 36 − 20	5.45	121
Medial orbitofrontal cortex	L	− 20 35 − 15	5.27	146

All regions are reported at *p* < 0.05 (FWE) at cluster level.

### Effect of duration on gray matter volume

The model for duration of olfactory loss showed no effect of duration group on gray matter volume within the a priori defined ROIs. When duration of olfactory loss was added as covariate to the model for the effect of etiology on gray matter volume for an exploratory analysis, this model displayed four significant clusters: the left (MNI (− 11, 26, − 20), Z > 8.00) and right (MNI(− 12, 24, − 17), Z = 6.19) gyrus rectus, and the left (MNI (− 20, 35, − 15), Z = 5.00) and right (MNI (20, 36, − 20), Z = 5.54) OFC, which were similar to the significant clusters found in the previous model. This indicates that adding duration of olfactory loss did not significantly affect the results of the model for etiology of olfactory loss.

### Association between gray matter volume and olfactory function

**Figure 2:**
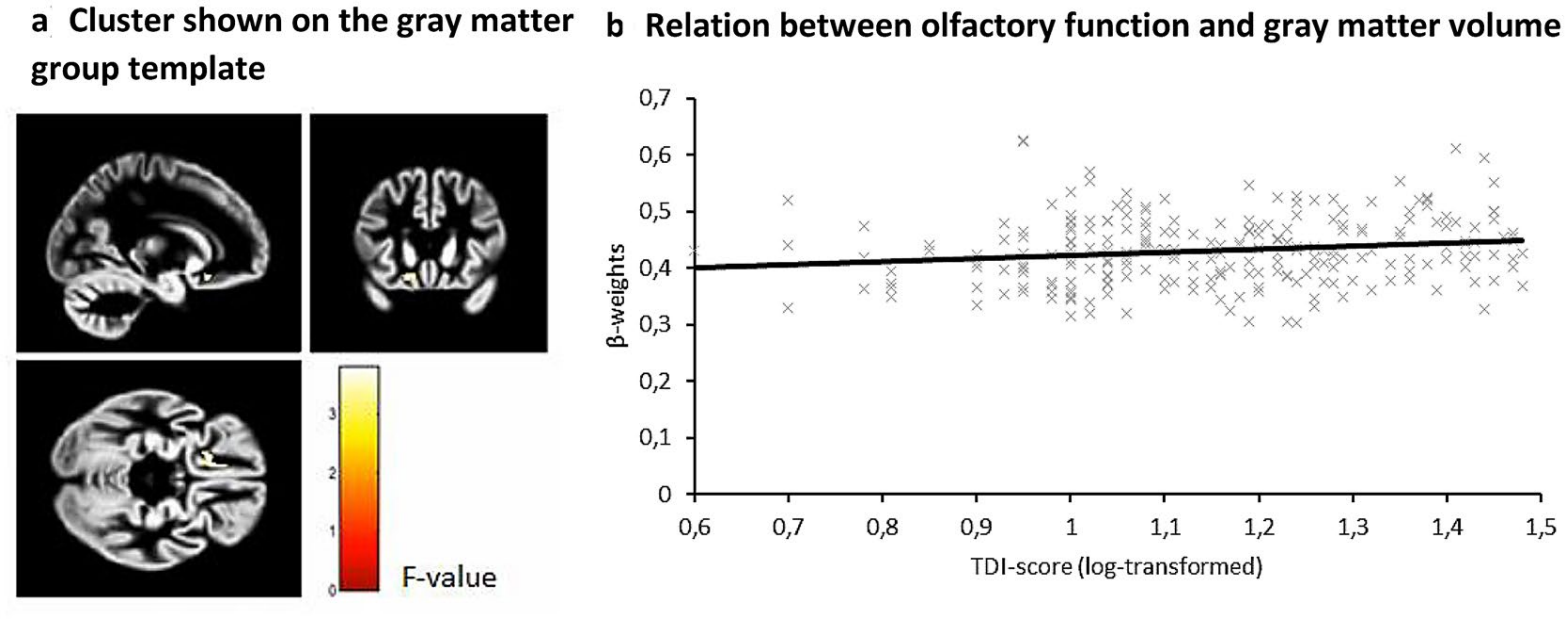
Association between olfactory function and mean gray matter volume in the left superior OFC cluster (peak MNI coordinates: − 18, 20, − 15), reported at *p* < 0.001; (**a**) shown on the gray matter group template; (**b**) relation between olfactory function and gray matter volume plotted.

TDI-score was used as a continuous variable to investigate the association between olfactory function and regional gray matter volume. There was a significant positive association between olfactory function and gray matter volume in the left superior OFC (MNI(− 18, 20, − 15), Z = 3.73, k = 193, r = 0.21) (Fig. 2).

## Discussion

We investigated morphological changes in olfaction-related brain regions in patients with olfactory loss by different etiologies and varying in duration. Etiology of olfactory loss significantly affected the gray matter volume of clusters in the gyrus rectus and the orbitofrontal cortex, while there was no effect of duration of olfactory loss. Patients with congenital anosmia had increased gray matter volume in parts of the medial and superior OFC, while gray matter volume of the gyrus rectus was decreased compared to the other patient groups. Olfactory function was significantly associated with gray matter volume of the left superior OFC.

The gray matter volume of the gyrus rectus was reduced in our patients with congenital anosmia compared to the other patient groups. Previous studies showed increased gray matter volume in the gyrus rectus in perfumers, who have trained their olfactory function, compared to healthy controls. Moreover, increased gray matter volume in the left gyrus rectus was associated with a longer duration of training^31^. This concurs with our finding that patients who were never able to smell have decreased gray matter volume in this region. This finding also dovetails with the reduced GM density of the gyrus rectus seen in patients with anosmia after traumatic brain injury^21^ and due to chronic rhinosinusitis^22^ compared to healthy controls; both studies did not find this reduction in gray matter density in hyposmic patients, who had a better olfactory function^21,22^. Altogether, this reduction in gray matter volume in the gyrus rectus points towards an important role of the gyrus rectus in olfactory function which may depend on the degree of exposure to olfactory input.

The OFC is known to contain secondary olfactory cortex^32^ and is involved in for example odor identification^33^ and hedonic processing of odors^34^. In the current patient population, a significant relation between TDI-score and gray matter volume was shown in the left superior OFC. In contrast, others found a positive correlation between TDI-scores and gray matter volume of the right OFC in healthy individuals^10,35^. Additionally, Bitter el al. demonstrated that volume of the right OFC was significantly decreased in anosmic^18^ and hyposmic^17^ patients compared to healthy controls, just as bilateral reduction in gray matter volume in the medial OFC in patients with olfactory loss after traumatic brain injury^21^ and in the right medial OFC due to chronic rhinosinusitis^22^. We conclude that gray matter volume in the OFC is associated with olfactory function in patients with olfactory loss. However, results on the location within the OFC from literature are conflicting with our results, warranting further research on the exact mechanism behind this association.

Patients with congenital anosmia, who had poorest olfactory function, showed an increased gray matter volume of the medial and superior OFC compared to the other patients. These results are in line with studies that found an increase in cortical thickness of the medial OFC^29^ and increased gray matter volume in the medial orbital gyrus bilaterally^30^ in patients with congenital anosmia compared to healthy controls. However, they contrast with Karstensen et al., who showed a reduced gray matter volume of the medial OFC in patients with congenital anosmia^28^. While results are conflicting, they all suggest that congenital anosmia may give rise to volumetric changes in the OFC. Overall, further research is needed to better understand the effect of congenital anosmia on gray matter volume of the medial OFC. The mechanism behind this effect might be fundamentally different than found in patients with acquired olfactory loss, as patients with congenital anosmia were never able to smell throughout their whole life. In these patients, structure probably drives function instead of the other way around.

Neuroanatomical changes in patients with olfactory loss have so far mainly been studied with regard to etiology of olfactory loss. Our results suggest that the effect of etiology of olfactory loss on volume of olfaction-related brain areas is not mediated by duration of olfactory loss, as effects of etiology and duration were consistently apparent in the same brain regions, namely the gyrus rectus and orbitofrontal cortex. However, we did see that olfactory function decreased over time: patients with a longer duration of olfactory loss had the lowest scores on the Sniffin’ Sticks, in line with previous findings^6^. We postulate that morphological changes in the OFC and gyrus rectus following acquired olfactory loss might be due to changes in the degree of exposure to olfactory input.

Our unique collaboration with the Smell and Taste Center in Ede allowed us to include a large population of clinically diagnosed patients (n = 257), whereas other studies only included smaller and inhomogeneous samples of patients (e.g.^18^(n = 17) and^20^(n = 19)) or only a specific group of patients (e.g.^22^(n = 21) and^19^(n = 16)). This allowed us to investigate etiology and duration of olfactory loss within the patient population. As there was no healthy control group in the current study, we chose to focus on regions in the brain that are known to play a role in olfactory processing^41^, which allowed us to compare results with literature. While previous studies also reported changes in other olfactory brain regions like the piriform cortex, the anterior cingulate cortex and the insular cortex (see e.g.^17–22^), no significant changes were found in these brain regions in the current study, despite our large sample size. Possibly, changes in these brain regions occur regardless of etiology or duration of olfactory loss or olfactory function. In a follow-up, inclusion of a group of healthy control participants would be warranted to further investigate morphological changes in patients with olfactory loss. Moreover, to specifically investigate morphological changes in patients with congenital anosmia a control group of participants similar in age should be included, as in our study the patients in this group were significantly younger than the patients in the other groups.

In the current study, patients with olfactory loss after head trauma were excluded, as this can lead to morphological changes unrelated to the olfactory loss per se. Han et al. found decreased volume in the gyrus rectus and the OFC of patients with olfactory loss compared to healthy controls as well as a higher degree of gray matter reduction in patients with anosmia compared to patients with hyposmia^21^, both consistent with our results. This shows that our current findings might also be generalized to a more diverse population of patients with olfactory loss. However, analysis of brain lesions in patients with olfactory loss after traumatic brain injury showed that 22 out of 41 patients had lesions in the OFC^21^. This indicates that morphological changes in olfaction-related brain regions in patients with olfactory loss after traumatic brain injury should be interpreted with care.

All together our results show that morphological changes in olfaction-related brain regions related to olfactory loss are reflected in gray matter volume of the gyrus rectus and the OFC. We suggest that these changes do not depend on etiology or duration of olfactory loss, but rather on olfactory function and the subsequent degree of exposure to olfactory input, in patients who lost their sense of smell at a later age. Patients with congenital anosmia showed an increased gray matter volume of the OFC compared to patients with acquired olfactory loss, which might be a result of the lack of exposure to olfactory input throughout life. Our results confirm the vital role of the OFC in olfaction.

## Methods

### Selection of patients

The patient population consisted of 369 patients who were clinically diagnosed with olfactory loss and visited the Smell and Taste Center in Hospital Gelderse Vallei (Ede, the Netherlands) between August 2015 and December 2018. All patients underwent an extensive testing protocol upon their visit, including testing of orthonasal and retronasal smell function and taste function. Only testing results relevant for the current study are described in this article.

Patient data were included for analysis in the current study when patients: were diagnosed with functional anosmia or hyposmia by an ENT-physician; were diagnosed with a quantitative smell disorder; had olfactory loss due to one of the following etiologies: post-infectious; chronic rhinosinusitis; idiopathic; or congenital anosmia. In total, 257 patients were included for analysis. Duration of olfactory loss was divided into five categories: 0–2 years; 2–5 years; 5–10 years; > 10 years; and congenital (‘never been able to smell’).

Patients included in this study all signed an informed consent on the use of their patient data for research. The current study met the requirements of the Declaration of Helsinki and was approved by the review committee for scientific research of Hospital Gelderse Vallei, Ede, the Netherlands (BC/1703–143).

### Measurement of olfactory function

Patients’ olfactory function was measured using the Sniffin’ Sticks^42^. This test measures odor threshold with *n*-butanol (T, score: 1–16), discrimination ability (D, score: 0–16) and identification ability (I, score: 0–16). The scores of the three separate parts of the test are summed up to a composite TDI-score (score: 1–48) and were used to categorized patients into functional anosmic (TDI ≤ 16); hyposmic (16 > TDI < 30.75) or normosmic (TDI ≥ 30.75)^43^. For 10 out of 257 patients, TDI-score could not be obtained due to missing results.

### Image acquisition and voxel-based morphometry preprocessing

MRI data was acquired on a 3 T Siemens Magnetom Verio scanner (Siemens, Erlangen, Germany). Using a 32-channel head coil, a 1-mm isotropic sagittal T_1_-weighted 2D MP-RAGE scan was made (TE/TR: 2.26/1900 ms; flip angle: 9°; 192 slices FoV: 256 × 256 mm).

Voxel-based morphometry (VBM) analysis was done using the Computational Anatomy Toolbox (CAT12, r1363) implemented in SPM12 (v7219, Wellcome Centre for Human Neuroimaging, UCL, London, UK) and executed in Matlab R2018 (The Mathworks, Natick, MA, USA) by following the CAT12 manual (http://dbm.neuro.uni-jena.de/cat/index.html). All images were manually reoriented to the same point of origin by using the Montreal Neurological Institute (MNI) template as a reference. Spatial normalization and tissue classification were performed by using the default settings in CAT12, including the standard tissue probability maps in SMP12^44^. The T_1_-weighted images were segmented into gray matter (GM), white matter (WM) and cerebrospinal fluid (CSF). Voxels were resliced to 1.5 × 1.5 × 1.5 mm and total intracranial volume (TIV), GM volume and WM volume were calculated.

The modulated gray matter images were used to check the sample homogeneity in CAT12, using TIV as a covariate. There was a large homogeneity (r > 0.85). Scans that were indicated as outliers in the boxplot were manually inspected for inhomogeneity due to movement artefacts; this was done for 13 scans. None of these scans were excluded due to poor image quality.

As a next step, the GM and WM segmentations were used to create a group-specific probabilistic template in the Diffeomorphic Anatomical Registration through Exponentiated Lie algebra (DARTEL) toolbox to refine inter-subject registration^45^. Finally, the modulated GM and WM segmentations were smoothed with the default settings, using an isotropic 3D Gaussian kernel with a full width at half maximum of 8 mm.

The effect of etiology and duration on regional gray matter volume was tested using a region of interest (ROI) analysis including a priori ROIs in both primary and secondary olfaction-related brain regions that are known to play a role in olfactory processing from meta-analyses^13,41^. The Automated Anatomic Labeling (AAL) atlas^46^ in the WFU pickatlas toolbox^47^ was used to create an ROI mask containing the following regions (bilaterally): anterior cingulate cortex (ACC), amygdala, caudate, hippocampus, insula, inferior OFC, medial OFC, superior OFC, piriform cortex, entorhinal cortex, parahippocampal gyrus, putamen, gyrus rectus and superior temporal pole. While the gyrus rectus surrounds the olfactory sulcus, it is not commonly acknowledged as olfactory region. It is mostly related to cognitive processes, such as memory recall^48,49^. However, it has been shown to be involved in both anatomical and functional olfactory networks^41^ and was therefore included as a region of interest.

### Analysis of patient characteristics

Analyses were performed with IBM SPSS Statistics (version 25). Results were considered significant at *p* < 0.05. Normality of the data was checked with the Shapiro–Wilk test. All data are reported as mean ± SD or N (%) unless mentioned otherwise.

For etiology, all patients were divided into 4 groups based on cause of olfactory loss. For duration, only patients with acquired olfactory loss were included. These patients were divided into 4 groups based on duration of olfactory loss, as described in Sect. 2.1. Groups were compared for age, Sniffin’ Sticks and total gray matter volume score by means of a Kruskal Wallis test, as the data were not normally distributed. When significant differences were found, the Dunn-Bonferroni procedure was applied for post-hoc pairwise comparisons.

To determine differences in total gray matter volume between the etiology or duration groups, a one-way ANCOVA was used, with total gray matter volume as dependent variable, and etiology or duration as fixed factor. Age, sex and TIV were added as covariates. When significant results were found, the Dunn-Bonferroni procedure was applied for post-hoc pairwise comparisons.

### MRI data analysis

Two full factorial models were used to test the effect of etiology and duration of olfactory loss on gray matter volume. In the model for etiology, all patients were included. In the model for duration, patients with congenital anosmia were excluded, as this group was similar in both models. An additional, exploratory, full factorial model with etiology as factor and duration as covariate was designed to determine whether duration of olfactory loss would be a confounder in the results for etiology of olfactory loss. In this model, patients with congenital anosmia were included in the duration covariate.

In all models, sex, age and TIV were added as covariates. Covariates were mean centered, and an absolute threshold of 0.2 was applied to ensure analysis was restricted to gray matter voxels only. A threshold of *p* < 0.05 family-wise error-corrected at the cluster level was employed. The MarsBaR toolbox^50^ was used to extract mean gray matter volume in significant clusters based on the ROI analysis for each patient. These volumes were used for post-hoc testing to get more insight in the differences between the etiology groups, taking overfitting into account^51^. Post-hoc testing was done in SPSS by comparing mean gray matter volume in significant clusters between the different groups for etiology using Mann–Whitney *U* tests.

Additionally, a multiple regression model including TDI-score as a covariate was used to test the association between olfactory function and regional gray matter volume. As TDI-scores were not normally distributed, the scores were log-transformed. Analysis at *p* < 0.05 (FWE) at cluster level yielded no significant results. Therefore, we performed an exploratory analysis using *p* < 0.001 (uncorrected) with a cluster extent threshold of k > 159 voxels, based on the expected number of voxels per cluster (as described in the CAT12 manual).
